# Risk factors associated with academic difficulty in an Australian regionally located medical school

**DOI:** 10.1186/s12909-017-1095-9

**Published:** 2017-12-28

**Authors:** Bunmi S. Malau-Aduli, Teresa O’Connor, Robin A. Ray, Yolanda Kerlen, Michelle Bellingan, Peta-Ann Teague

**Affiliations:** College of Medicine and Dentistry, James Cook University, QLD, Townsville, Australia

**Keywords:** Academic difficulty, Medical students, Regionally located medical school, Risk factors

## Abstract

**Background:**

Despite the highly selective admission processes utilised by medical schools, a significant cohort of medical students still face academic difficulties and are at a higher risk of delayed graduation or outright dismissal.

**Methods:**

This study used survival analysis to identify the non-academic and academic risk factors (and their relative risks) associated with academic difficulty at a regionally located medical school. Retrospective non-academic and academic entry data for all medical students who were enrolled at the time of the study (2009–2014) were collated and analysed. Non-academic variables included age at commencement of studies, gender, Indigenous status, origin, first in family to go to University (FIF), non-English speaking background (NESB), socio-economic status (SES) and rurality expressed as Australian Standard Geographical Classification-Remoteness Area (ASGC-RA). Academic variables included tertiary entrance exam score expressed as overall position (OP) and interview score. In addition, post-entry mid- and end-of-year summative assessment data in the first and second years of study were collated.

**Results:**

The results of the survival analysis indicated that FIF, Indigenous and very remote backgrounds, as well as low post-entry Year 1 (final) and Year 2 (mid-year and final) examination scores were strong risk factors associated with academic difficulty. A high proportion of the FIF students who experienced academic difficulty eventually failed and exited the medical program. Further exploratory research will be required to identify the specific needs of this group of students in order to develop appropriate and targeted academic support programs for them.

**Conclusions:**

This study has highlighted the need for medical schools to be proactive in establishing support interventions/strategies earlier rather than later, for students experiencing academic difficulty because, the earlier such students can be flagged, the more likely they are able to obtain positive academic outcomes.

## Background

Medical schools world-wide have an obligatory mandate to tailor their educational training programmes to meet the health needs of their populations [1, 2], hence the recent University trend of adjusting the selection criteria for prospective medical students from rural areas. Based on the likelihood of a greater rate of return to rural regions as general practitioners, a number of Australian medical schools have engaged an inbuilt screening system to encourage the entry of medical students from regional and rural areas of workforce need [3, 4].

James Cook University’s (JCU) College of Medicine was established in 2000 to address the workforce needs of under-served populations in Northern Australia. The major mandate was to produce medical graduates with outstanding attributes and competencies relevant to the health of Indigenous (First Nation) Australian communities - rural, remote and tropical medicine [5]. This core mandate resulted in the design of a selection process that reflects this strategic vision and appreciates the importance of attributes such as values and personal characteristics other than academic ability alone; with about 50% of new students from rural areas yearly [6, 7]. This selection method has widened access to the medical degree program for a diverse group of applicants, who previously would not have been selected or even considered applying in the first place [8].

These all-inclusive equity pre-entry initiatives are not limited to Australia alone; they have also been adopted in many other countries [9–11]. However, there is paucity in, and scanty published literature on the risk factors associated with academic difficulty within programs based on all-inclusive equity initiatives. Identifying the risk factors among these groups will ultimately aid the development of effective support systems and foster high retention rates through to graduation.

Several studies have identified a variety of criteria that serve as robust academic and non-academic predictors of academic success for selecting potential students [12–14]. Despite the rigorous implementation of these criteria in highly selective admission processes, researchers have reported that a significant cohort of medical students still face academic difficulties and are at a higher risk of delayed graduation or outright dismissal [15, 16]. Shulruf et al. [17], reported the inability of interview/admission test scores to predict subsequent student failure or drop-out and concluded that it might be more useful to focus on post-enrolment factors. However, other researchers have suggested the value of test scores and Grade Point Averages (GPA) on unimpeded progress towards graduation from medical school [18]. Non-academic factors such as gender, age, ethnicity, parent education and social class have also been reported to affect attrition rates in medical schools. However, outcomes of the published reports are inconsistent [19–29]. For instance, ethnicity was found to be a significant predictor of academic difficulty [25], but had no significant impact on attrition [21, 24, 30].

Given the significant financial and human resource investment by all stakeholders in medical training, it is important to look at factors that might help identify students who may not be well prepared for the rigours of medical education so that appropriate resources can be channelled early on in the process, to improve their chances of surviving their chosen career. This is particularly important because medical schools are required on ethical grounds, to be socially accountable to the needs of the communities and healthcare systems that they serve [31]. Only a few studies in Australia have considered the predictors of academic difficulty or attrition rates [29, 32, 33]. To the best of our current knowledge, no study has evaluated the risk factors associated with academic difficulty in a regionally located medical school like JCU. In view of this, we hypothesise that with the unique selection process, variables which have not been considered in previous studies may be significant risk factors for academic difficulty in a rural medical school setting. Additionally, we hypothesise that post-entry examination scores will be better predictors of academic difficulty than pre-entry admission and interview scores. Therefore, this study aims to identify the risk factors (non-academic and academic) associated with academic difficulty among students in the first of Australia’s regionally-located medical schools – JCU, and to provide an insight into the measure of their relative risk ratios and survival probabilities.

### The JCU medical program

James Cook University medical school is the first of Australia’s 14 rural clinical schools and was established in 2000 with a mandate to prepare and graduate doctors for practice in rural and remote Australia. Students from across Australia and overseas are selected from a range of backgrounds, including purposively recruiting students from rural areas, as rural background is positively associated with returning to rural locations as doctors [34]. The JCU medical school, like other regional medical schools, recruits students nationally, with about half of the student cohort from rural and remote settings. It also has a decentralised training program in which the majority of the curriculum is delivered in Townsville in the pre-clinical years (1–3); while in the clinical years (4–6), students are assigned to one of the four regional clinical school sites (Townsville, Mackay, Cairns and Darwin). However, unlike other medical schools that have a rural component added on to the curriculum, the JCU medical curriculum is infused with a culture of practice among underserved populations and altruism. Students undertake 20 weeks of rural or remote clinical practice across the 6 years of their degree.

The admission procedure involves a three-staged selection process: written application, tertiary entrance exam scores expressed as Overall Position (OP) score and a semi-structured interview. Interview panels consist of three members (a medical practitioner, an academic, and a community member) and applicants are assessed on skills and attributes such as self-reliance, motivation and communication.

The MBBS course is delivered in an integrated systems-based manner, with some clinical exposure from year one. The first three years of the course provide a systems-based introduction to the foundations of medicine, with early experiences in rural placements. The final three years of the programme comprise community teaching practices and small rural hospital-based rotations with the sixth year specifically designated as the pre-intern year.

Summative assessments comprise of Multiple Choice Questions (MCQs), Short Answer Questions (SAQs), Key Feature Problems (KFPs) and Objective Structured Clinical Examinations (OSCEs). Students are enrolled in 2 chained subjects for each academic year. A student either passes or fails the year, not individual modules. Students are allowed to fail one year of the program. If they fail a second year of the program they are excluded from the MBBS course.

### JCU’s current support program

JCU offers an active program of support to students enrolled in the first three years of the undergraduate medical program (MBBS). Students become members of a small home group (average size of 9 students) facilitated by experienced staff or senior students. This group meets weekly and students have an individual meeting with the facilitator within six weeks. Students with academic or personal difficulties are often identified within the first few weeks of the year through the home group program or the first invigilated test that students undergo during the first semester of study. These students are referred to the academic advisor.

Students with poor results have an individual meeting with the academic advisor to discuss their approach to study, their particular learning skills, and any challenges to their study. It is not unusual to identify significant personal issues during these meetings. When appropriate, these students are referred to support services both within and outside the university. Similarly, students who have unsatisfactory results in the mid-year exams are offered some specific support activities including the opportunity to review their exam papers with the academic advisor. The students are referred to the JCU Learning Advisors and are encouraged to attend a series of specific learning skills workshops developed for MBBS students.

Students who are repeating a year of the MBBS program also have a program of support including regular progress meetings with the academic advisor and identification of specific academic or personal problems that have contributed to their results with appropriate referral to support services. In addition, the students meet with the academic advisor as a group early in the year to discuss common challenges for repeating students and for mutual support.

## Methods

We collated retrospective non-academic and academic entry data for all medical students who were enrolled at the time of the study (2009–2014). All the non-academic variables which were obtained from students at the point of entry into the MBBS program and stored in the University Record System database were included in this study. These variables are age at commencement of studies, gender, Indigenous status, origin, first in family to go to University (FIF), non-English speaking background (NESB), socio-economic status (SES) and rurality status which is expressed as Australian Standard Geographical Classification-Remoteness Areas (ASGC-RA). ASGC-RA is categorised based on accessibility to a range of services - remoteness index. SES and ASGC-RA data for international students were not included in the analysis because they could not be verified. Academic data included the OP and interview scores. In addition, post-entry, mid-year and end of year summative assessment (expressed as average percentage scores of the multiple choice, short answer, key feature and OSCE components of the exam, for each student) data in the first and second years of study were collated. These data were extracted from the University’s Student Record System.

For the purposes of this study, students with academic difficulty were defined as those who had been required to repeat an entire year of study or those who had been required to take supplementary exams. Ethics approval for this study was obtained from the James Cook University Human Research Ethics Committee (HREC Project Number: H5802).

### Data analysis

The probability of academic difficulty at any time point during medical school (Years 1–6) was modelled using Survival analysis as described by Huff and Fang [23]. We used Survival analysis instead of Logistic Regression analysis because it uses a time-series continuous outcome variable (time) that serves to increase statistical power and provides more precise and absolute estimates of when academic difficulty was experienced in the medical school. In addition, more data can be used because unobserved academic difficulty times are included in the model. It also accounts for censored data. Censoring occurs when research subjects do not experience the event(s) of interest throughout the duration of the study [35]. Censored observations herein were the data for students who did not experience academic difficulty during the study period.

A Cox Proportional Hazards Model [36] in Survival analysis was used to estimate the relative risks of experiencing academic difficulty (hazard rate estimate). Hazard rate estimate is the dependent variable in the model and it is defined as the probability that a student with particular characteristics would experience academic difficulty at time t, given that the student had survived to that time [37]. A survival probability estimate was also calculated using the Kaplan-Meier method [38]. Survival curves and non-parametric statistical log rank test were used to determine significant differences between groups. The independent categorical variables in the model were Origin (domestic = 0, international = 1); Gender (male = 0, female = 1); FIF (Not first in family = 0, First in family = 1); Indigenous status (Non-Indigenous = 0, Indigenous = 1); NESB (Non-English speaking = 0, English speaking = 1); SES (low = 0, medium = 1, high = 2) and Rurality expressed as ASGC-RA (major cities = 0, inner regional = 1, outer regional = 2, remote = 3, very remote = 4). The numerical independent variables were age at entry into the program (17–47 years); OP scores (1 being the highest score achievable and 14 being the lowest score achievable); interview percent scores (41–100%) and post-Year 1 and 2 summative examination average percent scores (30–94%). The range of each variable is presented in parentheses. Each of the covariates has either a positive or a negative regression coefficient (B) value associated with it and this indicates the relationship between the particular covariate and the dependent variable. For the categorical variables, a positive B value means that the subgroup which is coded as “1” has a higher risk of academic difficulty; while a negative B value indicates that the subgroup coded as “0” has a higher risk of academic difficulty. For numerical variables, a positive B value indicates high risk of experiencing academic difficulty with an associated increase in the value of the variable, while a negative positive B value indicates that the risk is associated with a decrease in the value of the variable. Hazard ratio – Exp(B), which is the exponentiation of the regression coefficient, was used to measure the magnitude of the relationship between the individual covariates and the hazard-rate estimate (with the effects of all other variables held constant). Hazard ratio also allows for the comparison of the estimated risks of experiencing academic difficulty between subgroups of a particular variable. Hazard ratio values greater than 1.0 indicate greater risk; therefore to calculate the estimated risk of experiencing academic difficulty associated with a particular covariate, the difference between the estimated hazard ratio and 1.00 was examined [23]. Survival probability estimates were calculated for variables that showed significant effects and for subgroups within each variable of interest; the timings of their experiences of academic difficulty were estimated.

## Results

The student profile showed that 12.7% were international; 26% were FIF; 2.5% were Indigenous; 42% were males; 19.8% were from non-English speaking backgrounds; 22.9%, 61.5% and 15.6% were from low, medium and high socio-economic backgrounds respectively; while 59.5% were from outer regional (56%) and remote (3.5%) areas (Table 1). Of the total study sample (*n* = 1097), 178 students (16.22%) experienced academic difficulty. Of this number, 111 students experienced their first academic difficulty in year 1 (10.12%); 31 (2.83%) in year 2; 15 (1.37%) in year 3; 14 (1.28%) in year 4; 5 (0.46%) in year 5 and 2 (0.18%) in year 6. Fifty-six (5.1%) students experienced multiple academic difficulties (Table 2). The table also shows that 33 (3%) of this group of students failed and exited the program. The remaining study sample (919 = 83.8%) was censored, that means they did not experience academic difficulty.

**Table 1:** Profile of medical students

Characteristic	No of students	Frequency (%)
Age at commencement of study		
≤ 19	823	75
> 19	274	25
Origin		
Domestic	958	87.3
International	139	12.7
First in Family		
No	813	74
Yes	284	26
Indigenous Status		
Non-Indigenous	1070	97.5
Indigenous	27	2.5
Gender		
Male	462	42
Female	635	58
NESB		
English Speaking	922	80.2
Non-English Speaking	226	19.8
SES		
Low	251	22.9
Medium	675	61.5
High	171	15.6
ASGC		
Major cities	265	24.1
Inner Regional	180	16.4
Outer Regional	614	56.0
Remote	23	2.1
Very Remote	15	1.4

**Table 2:** Academic difficulty - Frequency of occurrence

Status	Variables	No of students	Percent
All forms of academic difficulties – students who passed/failed supplementary exams, failed and repeated year, failed and exited program	Yes	178	16.22
Multiple Difficulties – students who experienced academic difficulty more than once	Yes	56	5.10
Failed and exited – students who failed and exited the MBBS program	Yes	33	3.01
Year of initial academic difficulty	Year 1	111	10.12
	Year 2	31	2.83
	Year 3	15	1.37
	Year 4	14	1.28
	Year 5	5	0.46
	Year 6	2	0.18

The results of the Cox proportional-hazards regression for non-academic and academic variables are presented in Table 3. Of all the non-academic variables examined, only FIF (*p* = 0.015), Indigenous status (*p* = 0.0001) and ASGC-RA status (*p* = 0.009) were statistically significant risk factors associated with academic difficulty. The B values for FIF, Indigenous and ASGC-RA status were positive, with estimated hazard ratios Exp(B) of 1.66, 3.49 and 1.36, respectively. These ratios imply that students who were first in their family to go to university were 1.7 times more likely to experience academic difficulty than those who were not; Indigenous students were 3.5 times more likely to experience academic difficulty than non-Indigenous students; and students from very remote areas were 1.4 times more likely to experience academic difficulty than their counterparts from regional and major cities. There were also highly significant interaction effects between FIF*Indigenous (*p* = 0.003) and FIF*ASGC-RA (*p* = 0.000). Statistically significant effects of age, gender, origin, SES and NESB were not observed. The results also showed that 17 (49%) of the exiting students were FIF, while only 9.1% of these students were of Indigenous background.

**Table 3:** Risk ratios for non-academic and academic variables

Variable	df	Regression Coefficient (B)	Exp(B) - Hazard ratio	95% CI for Exp(B)		*p*-value
				Lower	Upper	
Age at start of study ≤19 = 0, >19 = 1)	1	−0.13	0.93	0.85	1.01	0.10
Origin (Domestic = 0, International = 1)	1	0.95	1.09	0.94	1.28	0.23
First in Family (No = 0, Yes = 1)	1	0.51	1.66	1.10	2.50	0.015
Gender (Male = 0, Female = 1)	1	−0.40	0.96	0.67	1.39	0.83
Indigenous Status (No = 0, Yes = 1)	1	1.25	3.49	2.02	6.17	0.0001
NESB (English = 0, Non-English = 1)	1	0.19	1.21	0.86	1.91	0.48
^a^SES	2					0.71
SES (1)	1	0.24	1.28	0.62	2.63	0.49
SES (2)	1	0.24	1.28	0.72	2.39	0.40
^b^ASGC-RA	4					0.000
ASGC-RA (1)	1	2.88	0.06	0.01	0.46	0.007
ASGC-RA (2)	1	1.84	0.16	0.02	1.22	0.077
ASGC-RA (3)	1	3.92	0.02	0.00	0.17	0.000
ASGC-RA (4)	1	2.26	0.10	0.00	1.69	0.112
FirstinFamily*Indigenous	1	1.09	2.96	1.46	6.00	0.003
FirstinFamily*ASGC-RA	1	0.64	1.89	1.34	2.67	0.000
OP Score	1	−0.53	0.58	0.175	1.97	0.389
Interview score	1	−0.04	0.95	0.65	1.40	0.812
Mid-Year 1 Exam	1	0.02	1.02	0.98	1.07	0.251
End-Year 1 Exam	1	−0.22	0.80	0.75	0.85	0.00
Mid-Year 2 Exam	1	−0.05	0.94	0.91	0.98	0.01
End-Year 2 Exam	1	−0.05	0.95	0.91	0.98	0.01

^a^SES (Low = 0, Medium = 1, High = 2)

^b^ASGC-RA (Major Cities = 0, Inner Regional = 1, Outer Regional = 2, Remote = 3, Very Remote = 4)

**Fig. 1:**
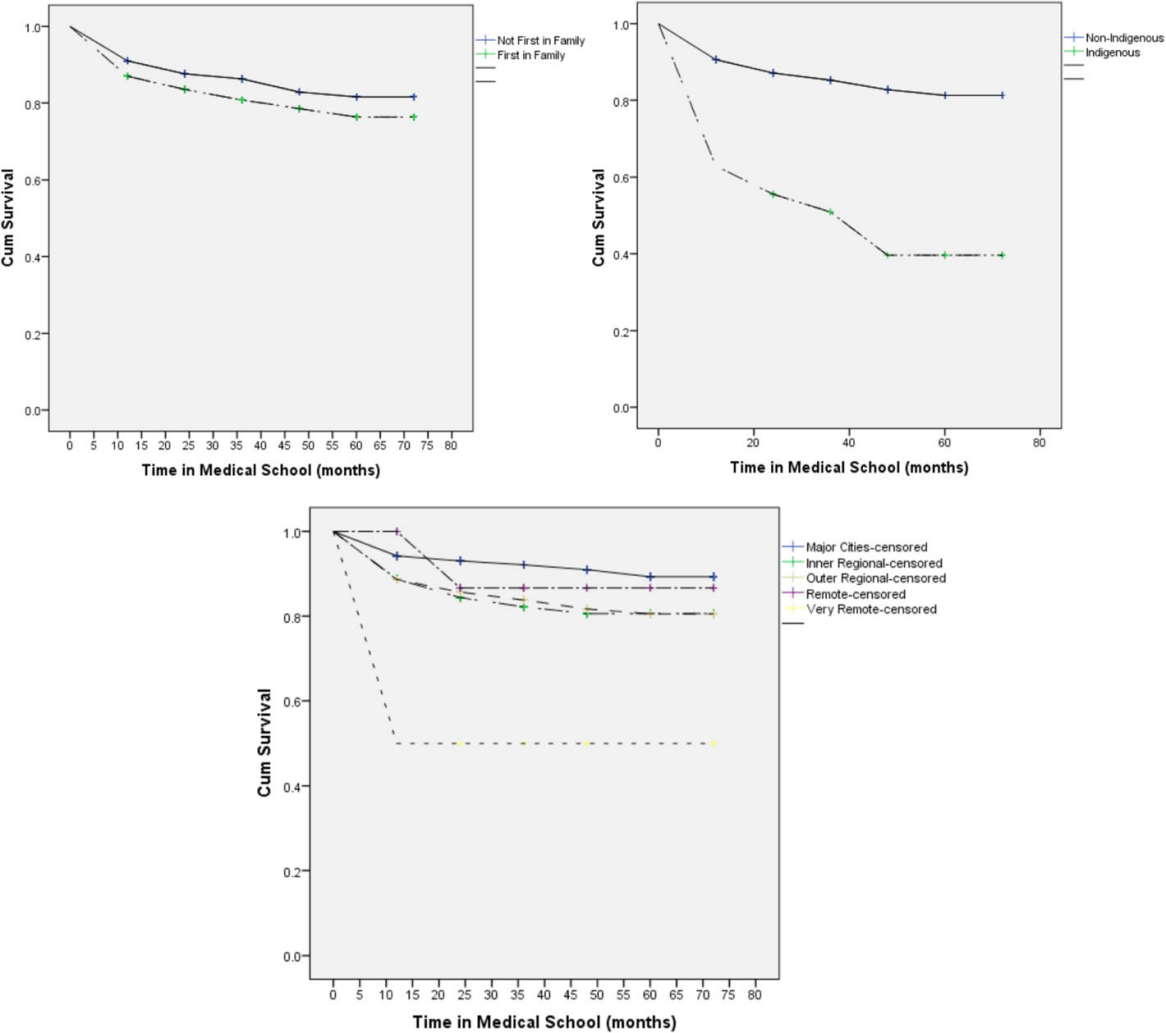
Survival Probabilities for sub-groups of risk predictors (Cumulative survival probability is the estimate of students who have not experienced academic difficulty during time (t) in medical school). **a** Survival Probabilities for First in Family Status. **b** Survival Probabilities for Indigenous Status. **c** Survival Probabilities for Rurality Status

Figure 1 portrays that overall survival probability estimates begin to drop at the end of the first academic year for all students, indicating that the first year of study is when students experience academic difficulty the most. The figure also shows that the decline is sharper and markedly increased for FIF and Indigenous students as well as for students from very remote areas in comparison to their respective counterparts. The results show that at the end of the sixth year of medical school, 75% of the FIF students had survived in comparison to 84%, of their non-FIF counterparts (Fig. 1a), 39% of the Indigenous students had survived in comparison to 83%, of the other students (Fig. 1b) and 40% of students from very remote areas had survived in comparison to 90% of their counterparts from major cities (Fig. 1c). Interestingly there was an 85% survival rate for students from remote areas in relation to 82% for students from outer and inner regional areas.

The results of the Cox proportional-hazards regression for the academic variables show that pre-entry OP and interview scores were not significant risk factors for academic difficulty, although the negative B values for both variables indicate that the risk of experiencing academic difficulty is associated with lower pre-entry OP and interview score. On the other hand, low post-entry Year 1 (final) and Year 2 (mid-year and final) examination scores were strong risk factors for academic difficulty. The B values for these exams were negative; with hazard ratios of 0.80, 0.94 and 0.95, respectively. This indicates that with increasing performance scores in the final year 1 exam, the risk of academic difficulty decreased by 20%, while it decreased by 6% and 5% for the Year 2 mid-year and final exams respectively. Interestingly, the mid-year exam in Year 1 was not a significant risk factor for academic difficulty.

## Discussion

We aimed to identify non-academic and academic risk factors for academic difficulty in an Australian regionally located medical school. FIF students were at a greater risk of academic difficulty during the course of their undergraduate medical program at JCU. This result corroborates the findings of Willoughby et al. [27], who reported significant effect of father’s highest educational level on student’s performance. We explored these findings further in our paper on dealing with academic difficulty in medical school, in which FIF students reported having deficiencies in strategic learning skills and poor academic integration as well as academic support deficits from their parents because their parents had little or no knowledge of university life and learning styles [39]. This was also alluded to by Thomson (cited in O’Neil et al. [40]) who purported that success as a university student requires an individual to possess a ‘social sense of direction’ or ‘habitus’ that allows him or her to navigate successfully within the prevailing educational culture of a programme. Thomson indicated that such skills are supposedly passed on by university-educated parents to their children, thereby making the latter more successful in higher education. Southgate and colleagues [8], in their study on the disadvantages and capacity to aspire to medical school also echo our findings. They found that FIF students had more limited opportunities to develop the types of strategic knowledge and practice that would help them navigate towards a career in medicine.

There is limited literature about factors that impact on the academic progression of Indigenous medical students, particularly in Australia [41]. Our study has demonstrated that Indigenous status constitutes a high risk factor for academic difficulty, indicating that Indigenous students were 3.49 times more likely to experience academic difficulty than their non-Indigenous counterparts. However, of the 33 students who failed and exited the program only 3 (9.1%) were Indigenous students. Additionally, none of the five (out of a total of 9) students from very remote areas who encountered academic difficulty exited the program. On the other hand, 18 (49%) of the exiting students were FIF students. This implies that even though Indigenous status is a high risk factor for academic difficulty, it does not often lead to exiting the medical program. This could be due to the extensive support provided at the university level to students from Indigenous and remote backgrounds and also because of the general cultural awareness and strong focus of the medical curriculum on rural, remote and tropical medicine and the health of Indigenous Australian communities. Very few successful support programs have been reported in the literature [42, 43]. Such programs have been developed, particularly to support students from educationally disadvantaged backgrounds to ensure that they receive optimal opportunities to succeed. Financial and academic assistance, personal support, inclusion of Indigenous health curriculum, provision of Indigenous role models and mentors are the major effective support resources that have used to foster the retention and successful progression of these students through their medical training [41–43].

There have been inconsistent findings on the effect of non-academic variables such as gender, SES, ethnicity, age at commencement and NESB on attrition [19, 21, 24–26, 30]. However, these variables were not significant risk factors for academic difficulty among students in this study. Specifically, Yates’ [25] finding that ethnicity was a significant predictor of academic difficulty, was not confirmed in this case.

This study has demonstrated that low academic scores in post-entry Year 1 and 2 summative examinations were high risk factors for academic difficulty, consistent with the findings of Hojat et al. [30]. For the 16.22% students who experienced academic difficulty, it mostly occurred in their first two years in the course, implying that successful completion of these 2 years reduces the risk of academic failure. The study also demonstrated the inability of pre-entry admission and interview test scores to predict academic difficulty. However, this result is incongruent with other studies that have reported pre-entry Medical College Admission Test (MCAT) and GPA scores as strong predictors of attrition [29, 40, 44]. It could be argued that the academic tasks required at high school level are very different to the tasks and assessment regime in the medical school. Therefore, it may be more useful to focus on post-enrolment factors to predict academic difficulty and/or failure in the medical school. Furthermore, an earlier study has demonstrated the advantage of the JCU model with particular emphasis on regionalised community capacity building, partnerships and placement opportunities in community teaching practices and small rural hospitals. The study indicated that although students from metropolitan backgrounds have higher quartile scores in years 1–3 than their peers from rural and remote settings, but by the time they get to years 4–6, which exposes them to rural clinical attachments, the academic performance become comparable [7].

While this study reflects the risk factors for academic difficulty at one medical school, the findings are generalisable, particularly to other regionally located medical schools that have engaged special selection systems to encourage the entry of medical students from regional and rural areas of workforce need. Such policies of widening participation have been shown to be highly relevant in this study, with the demonstrated high intake of students from the rural and remote settings – about half of the student cohort. Our study also shows that most of these students are not at risk of academic difficulty, confirming the findings of Ray et al. [7]. However, we have identified FIF, Indigenous students and those from very remote backgrounds as the most at-risk of academic difficulty in the medical school. Interestingly, a high proportion of the FIF students who experienced academic difficulty eventually failed and exited the medical program. As the majority of the student body at JCU are undergraduates mostly school leavers, the transition to tertiary education, particularly for those coming from smaller rural schools to a larger (in comparison) university presents its challenges. Studies indicate that students from disadvantaged backgrounds including rural and remote regions may be able to achieve rurally adjusted entry requirements for medicine; they often lack the educational opportunities to prepare themselves for tertiary education [45, 46]. This may be a possible explanation for the findings obtained in this study.

This study has also highlighted the need for medical schools to be proactive in establishing support interventions/strategies earlier rather than later, for students experiencing academic difficulty because, the earlier such students can be flagged, the more likely the students are able to obtain positive academic outcomes. Our experience at JCU indicate that retention of students from educationally disadvantaged backgrounds can be enhanced by providing specialised academic support programs that include mentoring and tutoring to nurture these students and help them develop effective time management and study skills. At JCU, student exposure to academic staff and peer support is enhanced through home group programs where groups of 8 or 9 students meet weekly with a senior student and a member of faculty to discuss and go through academic content material. We have also found that having an academic advisor, who is notified (by the year coordinator) and meets with students experiencing academic difficulty as soon as they are flagged, can enhance early activation of support mechanisms, rather than waiting for crisis intervention. Comments from student feedback systems suggest that these approaches have increased engagement with study and capacity to cope.

The promulgation of a culture of seeking support early is another important strategy that could be used as it may lead to fewer students experiencing extremes of academic difficulty. For this cultural change to succeed, it would require the active involvement of mentors, teaching staff and student groups in support services that aid early identification of marginally performing students so that tutorial assistance can be provided before they fail. A structured mentoring system could also be established to match students who are FIF or from rural and remote backgrounds with senior students and role models who can provide academic, emotional and social support. This approach can provide this group of students with a safer and more inclusive learning environment.

Overall, this study has identified the overriding importance of cultural issues as risk factors for academic difficulty (culture of higher education, indigenous culture and rural culture) as important areas for the development of proactive intervention and support programs for students with these backgrounds who are admitted into medical school. In addition, our other study indicated that when medical students are confronted with the possibility of academic failure, they ‘hide’ being unable to cope with the notion of failure and therefore find it hard to admit and share their problems with others [39].These results indicate that medical schools need to reconsider how they approach medical education for “at-risk” students. Further exploratory research will be required to establish effective intervention strategies that will aid this group of students in identifying and embracing their need for academic support.

### Study limitations

It is important to consider these results within the context of the study’s limitations. This study was conducted in only one medical school which offers an undergraduate entry program; hence cautious extrapolation of our findings to other medical schools with different selection methods and student profiles is needed to avoid sweeping generalisations. In addition, our coding for SES was collated by recorded students’ residential postcodes and this may not be representative of the true SES of the participants. Nevertheless, most of our findings have been confirmed in other studies, suggesting that our results are in tandem with and relevant for other medical schools, particularly those that have special mandates to recruit students from rural and remote settings in order to meet the health needs of the rural and remote communities they serve.

## Conclusions

This longitudinal retrospective study of 6 consecutive cohorts of students at JCU identified non-academic (first in family to go to university, Indigenous and rurality status) and academic (Year 1 and 2 summative examination scores) risk factors for academic difficulty. In addition, academic difficulty was mostly encountered in the first two years of study. The specific questions of “who” and “when” students are at risk of academic difficulty and attrition, should take centre stage in the quest for medical schools developing remediation and support programs for identifying at-risk students. Medical schools need to make maximum use of student data collected before and during the program to foster early identification of at-risk students. This approach will ultimately enable medical educators to provide every motivated student, irrespective of possible limiting backgrounds, with every opportunity to successfully cope with the rigours of medical school.

## Abbreviations

ASGC-RA: Australian Standard Geographical Classification-Remoteness Areas
FIF: First in family to attend university
JCU: James Cook University
MBBS: Bachelor of Medicine, Bachelor of Surgery
MCAT: Medical College Admission Test
MCQ: Multiple Choice Questions
NESB: Non-English speaking background
OP: Overall position
OSCE: Objective Structured Clinical Examination
SAQ: Short Answer Questions
SES: Socio-economic status

## References

[1] Boelen C (1999). Adapting health care institutions and medical schools to societies’ needs. Acad Med.

[2] Boursicot K, Roberts T (2009). Widening participation in medical education: challenging elitism and exclusion. High Educ Policy.

[3] Dunbabin JS, Levitt L. Rural origin and rural medical exposure: their impact on the rural and remote medical workforce. Rural Remote Health [internet] 2003;3:212. Epub 2003 Jun 25. 15877502

[4] Gale T, Parker S. Widening Participation in Australian Higher Education. Report to the Higher Education Funding Council of England (HEFCE) and the Office for Fair Access (OFFA), England. Leicester, UK: CFE (Research and Consulting) Ltd, 2013.

[5] Hays RB. New medical school for regional Australia. Med J Aust. 2000;172(8):362–3. 10.5694/j.1326-5377.2000.tb124006.x 10840486

[6] Hays RB, Bower AJ (2001). Modifying academic ranking of rural and remote medical school applicants. MJA.

[7] Ray RA, Woolley T, Sen Gupta T. James Cook University’s rurally orientated medical school selection process: quality graduates and positive workforce outcomes. Rural and Remote Health 15: 3424. (Online) 2015. Available: https://www.ncbi.nlm.nih.gov/pubmed/26442581. 26442581

[8] Southgate E, Kelly BJ, Symonds IM (2015). Disadvantage and the ‘capacity to aspire’ to medical school. Med Educ.

[9] Tekian A, Foley RP (1997). Why do medical students from under-represented minorities choose – or not choose – primary care careers?. Acad Med.

[10] Young ME, Razack S, Hanson MD, Slade S, Varpio L, Dore KL, McKnight D (2012). Calling for a broader conceptualisation of diversity: surface and deep diversity in four Canadian medical schools. Acad Med.

[11] Mathers J, Parry J (2009). Why are there so few working-class applicants to medical schools? Learning from the success stories. Med Educ.

[12] McKenzie K, Schweitzer R. Who succeed s at university? Factors predicting academic performance in first year Australian university students. Higher Educ Res. Dev. 2001;20:21–33.

[13] James D, Chilvers C (2001). Academic and non-academic predictors of success on the Nottingham undergraduate medical course 1970-1995. Med Educ.

[14] Holmes DC, Doering JV, Spector M (2008). Associations among predental credentials and measures of dental school achievement. J Dental Educ.

[15] Cleland J, Arnold R, Chesser A (2005). Failing finals is often a surprise for the student but not the teacher: identifying difficulties and supporting students with academic difficulties. Med Teach.

[16] Cleland J, Milne A, Sinclair H, Lee AJ (2008). Cohort study on predicting grades: is performance on early MBChB assessments predictive of later undergraduate grades?. Med Educ.

[17] Shulruf B, Poole P, Wang GY, Rudland J, Wilkinson T (2012). How well do selection tools predict performance later in a medical programme?. Adv Health Sci Educ.

[18] Dunleavy DM, Kroopnick MH, Dowd KW, Searcy CA, Zhao X (2013). The predictive validity of the MCAT exam in relation to academic performance through medical school: a national cohort study of 2001-2004 matriculants. Acad Med.

[19] Simpson KH, Budd K (1996). Medical student attrition: a 10-year survey in one medical school. Med Educ.

[20] Arulampalam W, Naylor R, Smith J (2004). Factors affecting the probability of first year medical students’ dropout in the UK: a logistic analysis for the intake cohorts of 1980–92. Med Educ.

[21] Arulampalam W, Naylor R, Smith JP (2007). Dropping out of medical school in the UK: explaining the changes over 10 years. Med Educ.

[22] Fogleman BY, Zwagg RV (1981). Demographic, situational, and scholastic factors in medical school attrition. South Med J.

[23] Huff KL, Fang D (1999). When are students most at risk of encountering academic difficulty? A study of the 1992 matriculants to US medical schools. Acad Med.

[24] Stetto JE, Gackstetter GD, Cruess DF, Hooper TI (2004). Variables associated with attrition from Uniformed Services University of the Health Sciences medical school. Mil Med.

[25] Yates J (2012). When did they leave and why? A retrospective case study of attrition on the Nottingham undergraduate medical course. BMC Med Educ.

[26] Yates J, James D (2007). Risk factors for poor performance on the undergraduate medical course: cohort study at Nottingham University. Med Educ.

[27] Willoughby TL, Arnold L, Calkins V (1981). Personal characteristics and achievements of medical students from urban and non-urban areas. J Med Educ.

[28] Strayhorn G (1999). Participation in a premedical summer programme for under-represented minority students as a predictor of academic performance in the first three years of medical school: two studies. Acad Med.

[29] Ward AM, Kamien M, Lopez DG (2004). Medical career choice and practice location: early factors predicting course completion, career choice and practice location. Med Educ.

[30] Hojat M, Gonnella JS, Erdmann JB, Veloski JJ (1996). The fate of medical students with different levels of knowledge: are the basic medical sciences relevant to physician competence?. Adv Health Sci Educ Theory Pract.

[31] Strasser R, Neusy A (2010). Context counts: training health workers in and for rural and remote areas. Bulletin of the World Health Organisation Oct.

[32] Neame RL, Powis DA, Bristow T (1992). Should medical students be selected only from recent school-leavers who have studied science?. Med Educ.

[33] Powis DA, Waring TC, Bristow T, O’Connell DL (1992). The structured interview as a tool for predicting premature withdrawal from medical school. Aust NZ J Med.

[34] Henry JA, Edwards BJ, Crotty B: Why do medical graduates choose rural careers? Rural and Remote Health 2009, 9:1083 (online). 19257797

[35] Singer JD, Willet JB (1993). It’s about time: using discrete-time survival analysis to study duration and timing of events. J Educ Stats.

[36] Cox DR (1972). Regression models and life tables. J R Stat Soc.

[37] Allison PD. Survival analysis using the SAS System: A Practical Guide. Cary, NC; SAS Institute, Inc. 1995.

[38] Collett D (2003). Modelling statistical data in medical research.

[39] O’Connor T, Malau-Aduli B.S, Ray R, Kerlen Y. Engaging students experiencing academic difficulty in the health profession courses. 2016 ANZAPHE conference, 19–23 March, Perth, Australia.

[40] O’Neill L, Wallstedt B, Eika B, Hartvigsen J (2011). Factors associated with dropout in medical education: a literature review. Med Educ.

[41] Garvey G, Rolfe IE, Pearson SA, Treloar C (2009). Indigenous Australian medical students’ perceptions of their medical school training. Med Educ.

[42] Acosta D, Olsen P (2006). Meeting the needs of regional minority groups: the University of Washington’s programmes to increase the American Indian and Alaskan native physician workforce. Acad Med.

[43] Spencer A, Young T, Williams S, Yan D, Horsfall S. Survey on Aboriginal issues within Canadian medical programmes. Med Educ. 2005;39(11):1101–9. 10.1111/j.1365-2929.2005.02316.x 16262805

[44] Urlings-Strop LC, Stijnen T, Themmen APN, Splinter TAW (2009). Selection of medical students: a controlled experiment. Med Educ.

[45] Chowdry H, Crawford C, Dearden L, Goodman A, Vignoles A. Widening participation in higher education: analysis using linked administrative data. Discussion paper no 4991. Volume discussion paper no 4991. Bonn: Institute for the Study of. Labour. 2010;

[46] Ray RA, Young L, Lindsay DB (2015). The influences of background on beginning medical students’ perceptions of rural medical practice. BMC Medical Education.

