# Correction to: Risk factors associated with academic difficulty in an Australian regionally located medical school

**DOI:** 10.1186/s12909-018-1170-x

**Published:** 2018-04-09

**Authors:** Bunmi S. Malau-Aduli, Teresa O’Connor, Robin A. Ray, Yolanda van der Kruk, Michelle Bellingan, Peta-Ann Teague

**Affiliations:** College of Medicine and Dentistry, James Cook University, Townsville, QLD Australia

## Correction

Following publication of the original article [1], one of the authors reported that prior to publication her surname had changed from ‘Kerlen’ to ‘van der Kruk’, but that this change had not been incorporated in the final version.

The original article has been updated.
